# Dynamics of *Clostridium* genus and hard-cheese spoiling *Clostridium* species in anaerobic digesters treating agricultural biomass

**DOI:** 10.1186/s13568-020-01040-4

**Published:** 2020-06-01

**Authors:** Alessandra Fontana, Mariangela Soldano, Paolo Bellassi, Claudio Fabbri, Francesco Gallucci, Lorenzo Morelli, Fabrizio Cappa

**Affiliations:** 1 Department for Sustainable Food Process – DiSTAS, Università Cattolica del Sacro Cuore, Via Emilia Parmense, 84, 29122 Piacenza, Italy; 2 Centro Ricerche Produzioni Animali – C.R.P.A. S.p.A., Viale Timavo, 43/2, 42121 Reggio Emilia, Italy; 3 Consiglio per la ricerca in agricoltura e l’analisi dell’economia agraria – CREA, Via della Pascolare, 16, Monterotondo, 00015 Rome, Italy; 4 Centro Ricerche Biotecnologiche, Università Cattolica del Sacro Cuore, Via Milano, 24, 26100 Cremona, Italy

**Keywords:** Anaerobic digestion, Pathogen, *Clostridium*, Agricultural biomass, Mesophilic, CSTR

## Abstract

Biogas plants are a widespread renewable energy technology. However, the use of digestate for agronomic purposes has often been a matter of concern. It is controversial whether biogas plants might harbor some pathogenic clostridial species, which represent a biological risk. Moreover, the inhabitance of *Clostridium* hard-cheese spoiling species in anaerobic digesters can be problematic for hard-cheese manufacturing industries, due to the issue of cheese blowing defects. This study investigated the effect of mesophilic anaerobic digestion processes on the *Clostridium* consortia distribution over time. Specifically, three lab-scale CSTRs treating agricultural biomass were characterized by considering both the whole microbial community and the cultivable clostridial spores. It is assessed an overall reduction of the *Clostridium* genus during the anaerobic digestion process. Moreover, it was evidenced a slight, but steady decrease of the cultivable clostridial spores, mainly represented by two pathogenic species, C*. perfringens* and *C. bifermentans*, and one hard-cheese spoiling species, *C. butyricum*. Thus, it is revealed an overall reduction of the clostridial population abundance after the mesophilic anaerobic digestion treatment of agricultural biomass. 
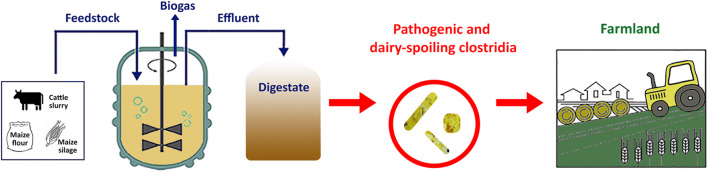

## Introduction

The number of anaerobic digestion (AD) plants is increasing, and the digestate by-product is generally distributed to farmlands for agronomic purposes. To this extent, we must consider the biological risks associated to the digestate spreading. Indeed, the biological process of AD cannot totally prevent the recontamination and regrowth of pathogenic bacteria. Most spore-forming pathogenic bacteria, such as *Clostridium* spp., are normally present in soil or animal feces; therefore, cattle manure or slurry sent to agricultural biogas plants can include spores of pathogenic bacteria (Bagge et al. 2005). Previous studies have indicated the reduction of some species of *Clostridium* after the AD process. Fröschle et al. (2015) highlighted the reduction of *C. botulinum* in mesophilic and thermophilic biogas digesters by means of microbial enrichment and Real-Time PCR techniques, indicating an improved sanitary quality of the digestion product compared to untreated substrates. However, they also observed a high persistence of the pathogenic *C. difficile* in manure-based AD treatments, whereas a minor relevance was given to the less abundant species of *C. chauvoei*, *C. haemolyticum*, *C. septicum* and *C. novyi* (Fröschle et al. 2015). Another research based on culture-dependent methods, evidenced the reduction of the pathogen indicator *C. perfringens* after mesophilic AD, in relation to the values of pH (8.3 ± 0.2) and toxic ammonia (2.9 ± 1.4 g kg^−1^) (Orzi et al. 2015).

In addition to the health risks related to the farming activity of spreading digestate as fertilizer, there are other concerns associated with the cheese-making process in Protected Designation of Origin (PDO) areas located in Northern Italy. Specifically, the presence of some species of *Clostridium* could be damaging for hard-cheese manufacturing industries which utilize local biogas plants for energy recovery. Indeed, three species of *Clostridium, namely C. tyrobutyricum*, *C. butyricum*, and *C. sporogenes,* are known to cause blowing defects in hard cheeses (Bassi et al. 2015). This issue causes cheese spoilage and thus, the loss of the final product with the related economic implications.

*Clostridium* spp. are known to be among the main players during the hydrolytic and acidogenic phases of AD (Fontana et al. 2016). Therefore, it is important to assess whether some clostridial species in the digestate from agricultural biogas plants might represent a biological risk considering both agronomic practices and food industry processes. Currently, the mechanisms of pathogenic decay via AD are not fully understood. Operating conditions strongly affect the relative abundance of pathogenic microorganisms by enriching or inhibiting them, depending on the species (Sahlström et al. 2008; Li et al. 2015; Ju et al. 2016). For instance, spore-forming species within the *Clostridium* and *Bacillus* genera are generally resistant to environmental stresses and high temperature conditions. Thus, they can survive thermophilic AD treatments (Dixit et al. 2005; Lloret et al. 2013). In addition to temperature, other factors affect the inactivation of pathogens. For example, the concentrations of ammonia and volatile fatty acids (VFAs), pH, and hydraulic retention time (Zhao and Liu 2019). However, many other AD variables play roles in the pathogenic decay or loss of diversity, such as the availability of nutrients and the operation modes of the reactors (e.g., batch or continuous) (Sahlström et al. 2008; Zhao and Liu 2019). Previous studies have provided contradictory data regarding the impact of AD on the *Clostridium* spp. It was generally reported that *Clostridium* consortia were not affected by the AD process (Bagge et al. 2005; Schnürer and Jarvis 2010). However, some case studies have detected a reduction in those consortia, depending on specific process conditions (e.g., low pH, high VFAs, high temperatures) (Bagge et al. 2005; Salsali et al. 2008; Orzi et al. 2015).

The present study performed an in-depth investigation about the effect of the mesophilic anaerobic digestion process on the *Clostridium* consortia, over time. Specifically, we evaluated the abundance of the *Clostridium* genus inside the digesters, by characterizing the whole microbial community via *16S rRNA* gene amplicon sequencing. Moreover, cultivable clostridial spores were identified at the species level by Sanger sequencing the complete *16S rRNA* gene. Thus, we revealed the impact of the mesophilic AD process on the spreading of both pathogenic and hard-cheese spoiling *Clostridium* spp., considering agricultural and dairy industry environments.

## Materials and methods

### Operating parameters of lab-scale reactors

Three 16 L lab-scale Continuous Stirred Tank Reactors (CSTRs), operating under the same conditions (biological replicates), were used to carry out anaerobic digestion tests (Giorni et al. 2018). The reactors were daily fed with a mixture commonly used in agricultural biogas plants, namely 45% cattle slurry, 10% maize flour and 45% maize silage, and run under mesophilic conditions (40 ± 0.2 °C). The hydraulic retention time (HRT) was 47 days and the organic loading rate (OLR) was 4 kg VS/m^3^ day. These process parameters were chosen in relation to the characteristics of the substrates (Additional file 1: Table S1). Tests lasted 3 months and started by introducing digestate in each reactor as *inoculum* taken from an agricultural biogas plant.

### Physico-chemical analyses

The pH was measured by a bench pH meter (XS instruments). Total solids (TS), volatile solids (VS), ammonium concentration (NH4^+^—N) and volatile fatty acids (VFAs), were measured as described in standard methods (APHA 2005). Total acidity and FOS/TAC were determined as described by Nordmann (1977) through an automatic titrator (TIM 840, Hach Lange). FOS represents the volatile organic acids content (mg/LHAc), while TAC stands for the total inorganic carbon (basic buffer capacity) (mg CaCO_3_/dm^3^). Methane content in the biogas was measured by a portable biogas analyzer (GA2000 PLUS, Geotechnical Instruments, UK). Determination of the inoculum methane yield as biochemical methane potential (BMP) was performed in accordance with the standard ISO 11734.

### DNA extraction for Illumina sequencing

DNA was extracted from 200 mg of sample using the Fast DNA™ SPIN Kit for Soil (MP Biomedicals, LLC, Solon, OH) according to the manufacturer’s protocol. DNA concentration was determined with the Quant-iT dsDNA HS assay kit and the Qubit fluorometer (Invitrogen, Carlsbad, CA, USA). The quality of the extracted DNA was checked with agarose gel electrophoresis, and then sent to the sequencing facility for *16S rRNA* gene amplicon sequencing (V3-V4 regions) using the Illumina MiSeq technology (2 × 300 bp). The microbial community composition of the three reactors was investigated in the inoculum and the reactor digestate at two time-points, representing the start-up phase (day 10) and steady-state condition (day 64).

### Bioinformatics and statistical analyses

Bioinformatics and statistics on the Illumina reads were performed with QIIME 2 2018.8 (Bolyen et al. 2018). Raw sequence data were demultiplexed and quality filtered using the q2‐demux plugin followed by denoising with DADA2 (Callahan et al. 2016). Alpha‐diversity metrics (observed OTUs, Shannon and evenness), beta-diversity metric unweighted UniFrac (Lozupone and Knight 2005) and PCoA were estimated using q2‐diversity after samples were rarefied (subsampled without replacement) to 30,000 sequences per sample. Taxonomy was assigned to OTUs using the q2‐feature‐classifier (Bokulich et al. 2018) classify‐sklearn naïve Bayes taxonomy classifier against the Greengenes 13_8 99% OTUs reference sequences (McDonald et al. 2012). Heat map representing genera relative abundances was visualized using MeV (Saeed et al. 2003). Statistical analyses to identify significance of changes in genera relative abundance between the inoculum, start-up and steady-state conditions, were carried out using STAMP software (Parks and Beiko 2010). Raw reads were deposited in Sequence Read Archive (SRA) database (BioProject accession number PRJNA602414).

### Clostridial spores cultivation and DNA extraction for Sanger sequencing

To identify the more resistant and abundant clostridia at species level, the full-length *16S rRNA* gene was sequenced using the Sanger technology. More specifically, the cultivable clostridia were quantified by counting the spores on Reinforced Clostridial Medium (RCM) supplemented with 0.2 g/L of cycloserine and 0.05 g/L of neutral red solution as described by Jonsson (1990). The spore counts were done for the inoculum, feedstock, and reactor digestate, at the start-up (day 10) and steady-state conditions (day 64). Approximately 10% of the grown colonies was isolated and the DNA was extracted with MicroLYSIS^®^ PLUS (Labogen, London, UK). Full-length *16S rRNA* gene was amplified using the primers P0 (5′-GAGAGTTTGATCCTGGCT-3′) and P6 (5′-CTACGGCTACCTTGTTAC-3′) (Di Cello and Fani 1996); the PCR product was then purified and sequenced. One-way ANOVA followed by Tukey’s multiple comparisons test on the microbiological count data were performed using GraphPad Prism version 5.

## Results

### Reactor performance

The physico-chemical characteristics of the inoculum and the digestates, along with reactor performance data, are reported in Table 1. The average methane yield of the three replicate reactors was 339.3 ± 1.4 Nm^3^ CH_4_/t VS at the steady-state condition, with 53.7 ± 0.3% of methane content in the biogas. The average pH value of the digestates was 7.70 ± 0.05 during processing in all three reactors, with an average FOS/TAC ratio of 0.28 ± 0.02. The total acidity was mainly due to acetate and butyrate in the inoculum, but acetate was the main VFA in the start-up and steady-state phases. Specifically, the acetate concentration decreased during the reactor start-up phase, then remaining constant until steady-state condition (Table 1). The same trend was observed for the ammonium concentration.

**Table 1:** Physico-chemical characteristics of the inoculum and the reactor digestate at the start-up and steady-state conditions

Sample	pH	Acetate (mg/kg)	Butyrate (mg/kg)	Total acidity (mg acetic acid eq/kg)	N-NH_4_^+^ (mg/kg)	FOS/TAC	CH_4_ (%)	CH_4_ yield (Nm^3^/t VS)
Inoculum	7.90 ± 0.02	123 ± 17	87 ± 3	175 ± 30	1833 ± 18	0.22 ± 0.02	60.4 ± 0.4^b^	67.0 ± 0.9^b^
Reactor start-up (days 0–9)	7.70 ± 0.02	89 ± 6	0^a^	89 ± 6	1740 ± 17	0.24 ± 0.02	55.9 ± 3.2	329. 2 ± 19.8
Reactor steady-state (days 10–74)	7.70 ± 0.05	82 ± 5	0^a^	82 ± 5	1715 ± 1	0.25 ± 0.02	53.7 ± 0.3	339.3 ± 1.4

^a^Below the 50 mg/kg detection limit

^b^Data obtained from BMP tests

### Microbial community diversity

**Fig. 1:**
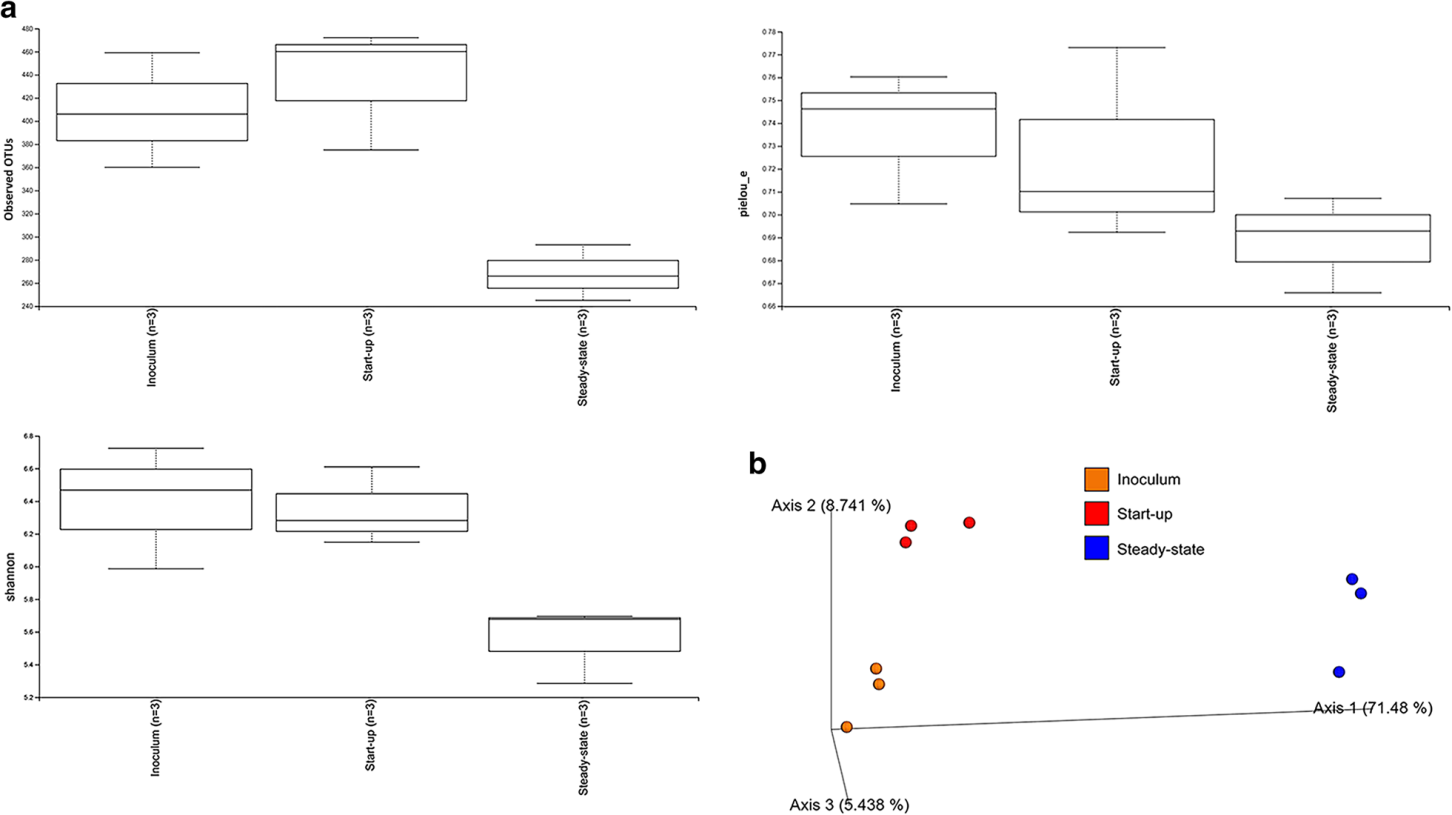
α-diversity plots. **a** Observed OTUs, Shannon index and Pielou’s index. **b** Principle coordinate analysis of the OTU distribution in the inoculum (orange dots) and in the three reactor replicates, during the start-up phase (red dots) and during steady-state conditions (blue dots)

We sequenced the *16S rRNA* gene amplicon to follow dynamic adaptations and selections within the entire microbial community in the inoculum and in the digestates at the reactor start-up and steady-state conditions. Microbial selection occurred at the OTU level, due to the digester operating conditions. Specifically, the α-diversity analysis indexes (i.e., observed OTUs, Shannon index, evenness) revealed a significantly different microbial community during the steady-state phase, compared to the initial inoculum and the start-up phase. In particular, the microbiota at the steady-state condition was less biodiverse (i.e., lower OTU richness) than at the start-up phase and the inoculum (Fig. 1a). In contrast, the OTU evenness (i.e., Pielou’s index) was not significantly different between time points. A principle coordinate analysis for testing β-diversity also highlighted the strong OTU composition changes between the inoculum, the start-up phase, and the steady-state condition (Fig. 1b).

### Microbial community taxonomy

**Fig. 2:**
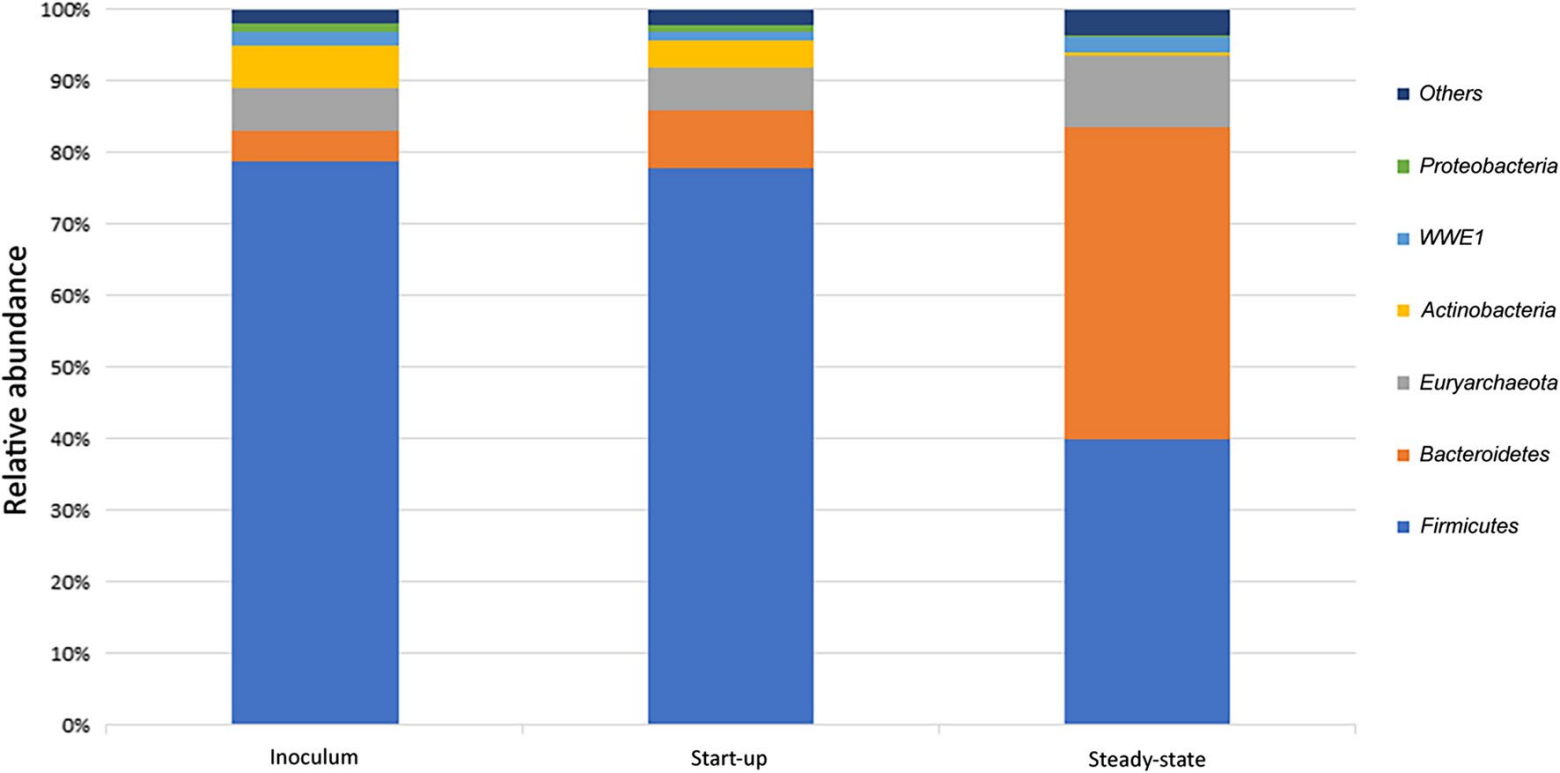
Taxonomy bar-plot shows the relative abundance of OTUs at the phylum level. Different OTU abundances are shown in the inoculum and in the reactor digestate, during start-up and steady-state conditions

**Fig. 3:**
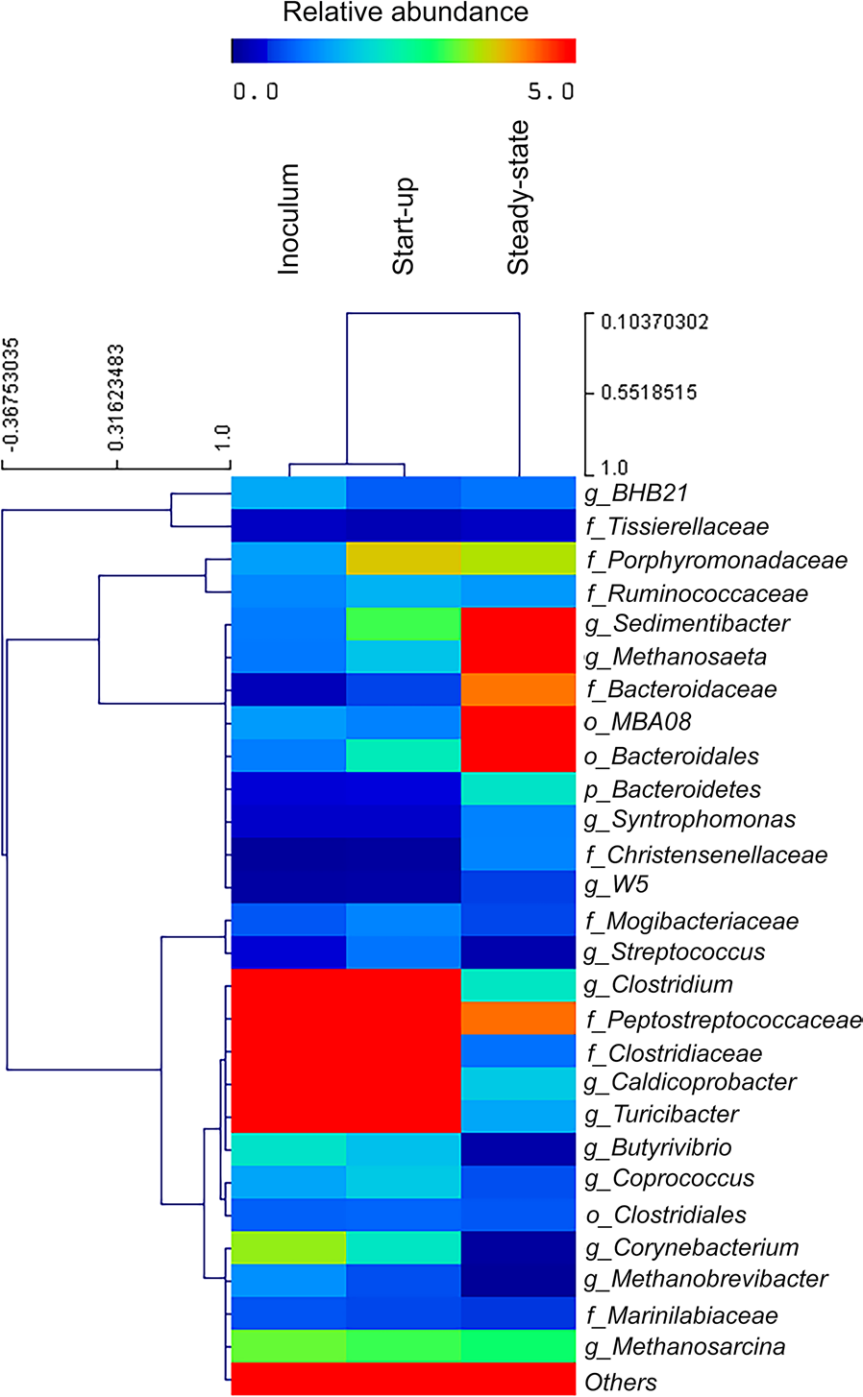
Heat map shows the relative abundance of the genera in the inoculum, the reactor start-up phase, and steady-state conditions. Only genera with relative abundances > 1% are represented

Taxonomic classification showed that the *Archaea* population (*Euryarchaeota* phylum) increased by an average of 4% at the steady-state, compared to both the inoculum and the start-up phase (Fig. 2). This increase was specifically observed in the genus *Methanosaeta*, which became the main archaea, along with *Methanosarcina*, at the steady-state. On the contrary, *Methanobrevibacter* genus, which accounted for 1.8% and 0.7% in the inoculum and at the start-up phase, respectively, nearly disappeared at the reactors’ steady-state (Fig. 3 and Additional file 1: Fig. S1).

**Fig. 4:**
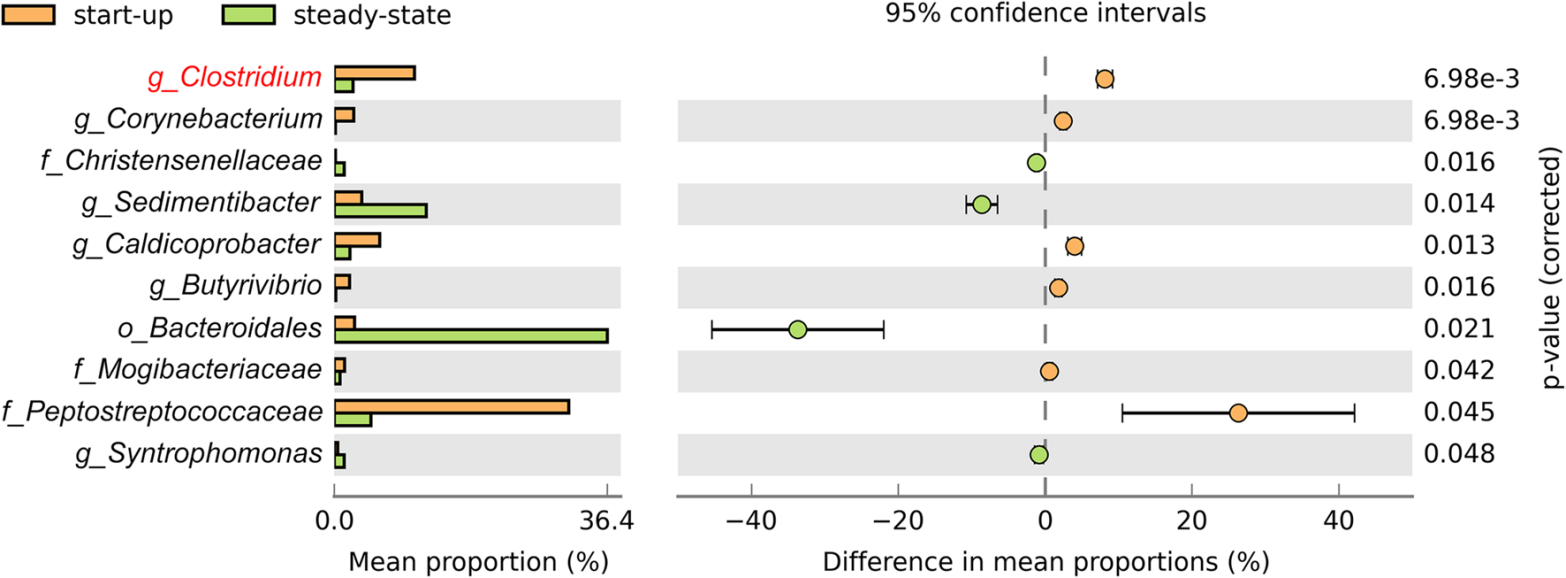
Genera that changed significantly (p < 0.05) during the steady-state, compared to the start-up phase. No statistically significant changes are observed between genera compositions in the start-up phase and the inoculum

Concerning the *Bacteria* population, the dominant phylum was *Firmicutes*, in both the inoculum and the reactor at the start-up phase (~ 80% relative abundance). The *Bacteroidetes* abundance ranged between 4% (inoculum) and 8% (start-up) (Fig. 2); however, during steady state, it reached ~ 45% relative abundance, whereas *Firmicutes* decreased to ~ 40% (Fig. 2). Within the *Firmicutes* phylum, the *Clostridium* genus strongly decreased, from a relative abundance of ~ 9% at the start-up phase, to ~ 2% at steady-state (Fig. 3 and Additional file 1: Fig. S1). Among the identified genera, *Clostridium* exhibited the most significant reduction (p < 0.05) in relative abundance (Fig. 4). However, a sharp reduction was also observed in the genera *Turicibacter*, *Caldicoprobacter*, *Butyrivibrio*, and *Coprococcus*. Conversely, the *Syntrophomonas* genus, which also belongs to *Firmicutes*, exhibited a sharp increase at the steady-state condition (Fig. 3 and S1). Considering the *Bacteroidetes* phylum, the main increase was observed in the *Bacteroidales* order (Fig. 3 and S1). Among the less abundant phyla, *Actinobacteria* (including the genera *Corynebacterium* and *Actinomyces*) and *Proteobacteria* nearly disappeared at the steady-state (Fig. 2). In contrast, the candidate phylum, *Waste Water of Evry 1* (*WWE1*) slightly increased (Fig. 2); within this phylum, two genera were evidenced: *BHB21* and *W5* (Fig. 3 and Additional file 1: Fig. S1).

### Identification of cultivable *Clostridium* spores

**Fig. 5:**
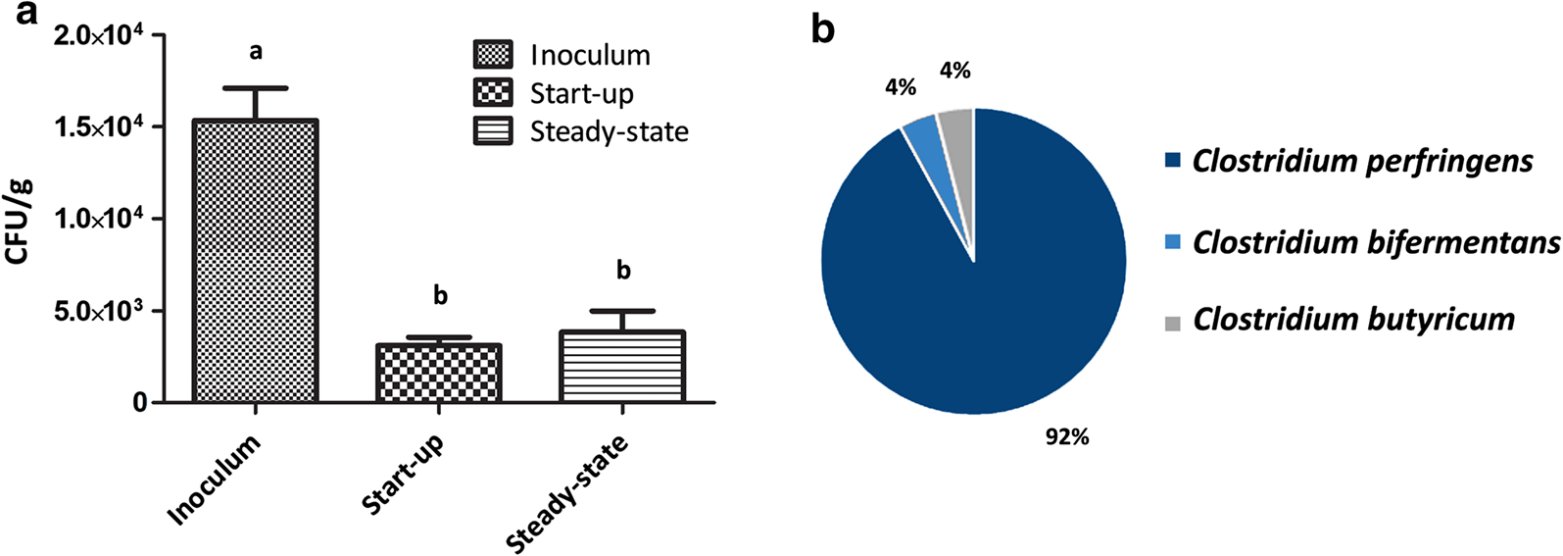
Cultivable *Clostridium* spores. **a** Spore counts in the inoculum and in the reactor digestate at start-up and in steady-state conditions. Letters in the graph indicate significant differences between samples, based on Tukey’s test. **b** Identification and relative abundance of the isolated *Clostridium* spores

This study was focused on the *Clostridium* population dynamics during the AD process. Therefore, we also evaluated the abundance and identity of the cultivable clostridial spores. Specifically, the inoculum contained 1.5 × 10^4^ CFU/g of spores, whereas the feedstock contained 2.1 × 10^3^ CFU/g of spores (mainly in the cattle slurry component). In the reactor digestate, the clostridial spore concentration slightly decreased to 3.1 × 10^3^ CFU/g at the start-up phase and remained at 3.9 × 10^3^ CFU/g at the steady-state condition (Fig. 5a). A one-way ANOVA followed by Tukey’s post hoc test showed that the spore contents were significantly different (p = 0.0007) between the inoculum and the reactor digestate, at both time points. Sanger sequencing revealed that the dominant species among the cultivated clostridia where *C. perfringens* (92%), *C. bifermentans* (4%), and *C. butyricum* (4%) (Fig. 5b).

## Discussion

Considering the reactor performance, both VFAs (mainly acetate) and ammonium compounds were not at concentrations that might inhibit the AD process (Chen et al. 2008; Fotidis et al. 2013), as also evidenced by the maintenance of the methane yield along the process. According to the characterization of the microbiota inside the reactors, it was experienced a microbial shift in terms of OTUs diversity at the steady-state, compared to the initial inoculum and the start-up phase. The decrease in biodiversity along the process was also pointed out in different studies (Lavergne et al. 2019; Cabezas et al. 2019; Castelló et al. 2011). This fact indicates that, in AD reactors, microbial diversity mainly relies on the inoculum. Moreover, the selection operated by the feed characteristics (i.e., pH, alkalinity, buffer capacity) on specific microbial consortia has been also pointed out (Fontana et al. 2018).

It is worth noting that the microbial composition change between the start-up and the steady-state condition did not affect the biogas production efficiency. The shift showed within the archaeal population indicated a variation of the undertaken methanogenic pathway at the steady-state: from hydrogenotrophic to acetoclastic. Indeed, at the taxonomic level, it was evidenced that the acetoclastic methanogenic genera *Methanosaeta* and *Methanosarcina* took over the hydrogenotrophic *Methanobrevibacter* genus. The selection of acetoclastic methanogens under mesophilic conditions was also observed in full-scale agricultural biogas plants (Campanaro et al. 2016; Fontana et al. 2016). This could be due to the presence of acetate as main VFA produced from the degradation of agricultural biomass.

On the other hand, the methane yield increase exhibited from the inoculum to the start-up phase may be reasonably due to the adaptation of the methanogenic consortia to the operating conditions of the reactors.

Regarding the *Bacteria* population, the dominance of *Firmicutes* and *Bacteroidetes* phyla was assessed in different AD systems for degrading various feedstocks (Fontana et al. 2016; Schlüter et al. 2008; Sundberg et al. 2013). Moreover, the main presence of *Bacteroidales* order within the *Bacteroidetes* phylum, was showed in most mesophilic AD processes with low concentrations of VFAs, total ammonia, and salt (Rivière et al. 2009; Nelson et al. 2011). Considering instead less abundant phyla, the increase in the *WWE1* candidate phylum over time was also described in a mixed plug-flow loop reactor fed with dairy manure (Li et al. 2014). This phylum includes members that can degrade cellulose substrates (Limam et al. 2014); thus, it might play a key role in full-scale CSTRs fed with maize-silage or other energy crops rich in cellulose. What is worth noting regards one among the main genera in AD, belonging to the *Firmicutes* phylum: *Clostridium*. Indeed, it was exhibited a sharp reduction in *Clostridium* spp. over time, from the inoculum and the start-up phase, to the reactor steady-state. This outcome contrasted with the results from Yergeau et al. (2016), who reported a significant increase in *Clostridium* DNA after the AD treatment.

Along with the *Clostridium* decrease, it was highlighted the increase in *Syntrophomonas* genus at the steady-state. Some species belonging to this genus were previously shown to be responsible for butyrate oxidation in AD (Treu et al. 2016; Zhao et al. 2018). Moreover, it has also been reported that some *Syntrophomonas* spp. could establish syntrophic associations with methanogens (e.g., *Syntrophomonas wolfei*) (Sieber et al. 2010). Thus, the low levels of butyrate (i.e., below the 50 mg/kg detection limit) in the system during the reactor steady-state might have been related to the increment in *Syntrophomonas* spp., along with the strong reduction in butyrate-producers, such as *Clostridium* spp. (Detman et al. 2019). The count of the cultivable clostridial spores confirmed the trend in relative abundance of the *Clostridium* genus observed with the amplicon sequencing approach. Thus, it is suggested that the mesophilic AD treatment caused a slight, but steady reduction also in the abundance of cultivable clostridial spores. Orzi et al. (2015) also reported the decrease of the pathogen indicator *C. perfringens* in mesophilic plants. However, we additionally revealed the presence of the pathogenic species *C. bifermentans* and the hard-cheese spoiling species *C. butyricum*, along with the dominant *C. perfringens*. The main presence of spores belonging to *C. perfringens* might be related to a higher competitiveness for carbon resources resulting in a better adaptation of the species to the mesophilic anaerobic digestion conditions (Orzi et al. 2015). However, the presence of *C. butyricum*, even if in lower abundance than the other cultivable spores, can suggest an issue of cross-contamination between local biogas plants and dairy facilities specialized in long-ripened cheese production.

To summarize, this study showed that the treatment of agricultural biomass via mesophilic anaerobic digestion has a reduction effect on the clostridial population at the genus level. Moreover, a slight, but steady decrease effect is also exhibited on the cultivable clostridial spores, which are mainly represented by *C. perfringens*, *C. bifermentans* and *C. butyricum*.

## Supplementary information

**Additional file 1: Table S1.** Characteristics of the substrates used as feedstock in the anaerobic digestion tests. **Fig. S1** Taxonomic assignments at the genus level. **a** Bar-plot of the OTU relative abundances in the inoculum and reactor digestate at the start-up and steady state conditions. **b** Widening of the minor genera.

## Data Availability

Raw reads were deposited in Sequence Read Archive (SRA) database (BioProject accession number PRJNA602414). Additional file 1.
