# Supplementary material for: Dynamics of Clostridium genus and hard-cheese spoiling Clostridium species in anaerobic digesters treating agricultural biomass
Source: AMB Express. 2020 Jun 1;10:102. doi: 10.1186/s13568-020-01040-4 (PMC7266885; doi:10.1186/s13568-020-01040-4)

Additional file 1

Dynamics of *Clostridium* genus and hard-cheese spoiling *Clostridium* species in anaerobic digesters treating agricultural biomass

Alessandra Fontana [1] ✉, Mariangela Soldano [2], Paolo Bellassi [1], Claudio Fabbri [2], Francesco Gallucci [3], Lorenzo Morelli [1,4] and Fabrizio Cappa [1,4]

[1] Department for Sustainable Food Process – DiSTAS, Università Cattolica del Sacro Cuore, Via Emilia Parmense, 84, 29122 Piacenza, Italy

[2] Centro Ricerche Produzioni Animali – C.R.P.A. S.p.A., Viale Timavo, 43/2, 42121 Reggio Emilia, Italy

[3] Consiglio per la ricerca in agricoltura e l'analisi dell'economia agraria – CREA, Via della Pascolare, 16, 00015 Monterotondo, Roma, Italy

[4] Centro Ricerche Biotecnologiche, Università Cattolica del Sacro Cuore, Via Milano, 24, 26100 Cremona, Italy

✉ Email: alessandra.fontana@unicatt.it

Table S1

Characteristics of the substrates used as feedstock in the anaerobic digestion tests

Substrate	pH	Total solids (TS)	Volatile solids (VS)	
		(g/kg)	(g/kg)	(%TS)
**Cattle slurry**	7.2 ± 0.3	74.2 ± 7.9	58.2 ± 4.6	78.7 ± 2.8
**Maize silage**	3.8 ± 0.5	336.7 ± 9.5	323.1 ± 11.0	95.9 ± 0.6
**Maize flour**	5.4 ± 0.4	935.4 ± 3.1	922.9 ± 4.2	98.7 ± 0.1

Fig. S1

Taxonomic assignments at the genus level. **a** Bar-plot of the OTU relative abundances in the inoculum and reactor digestate at the start-up and steady state conditions. **b** Widening of the minor genera

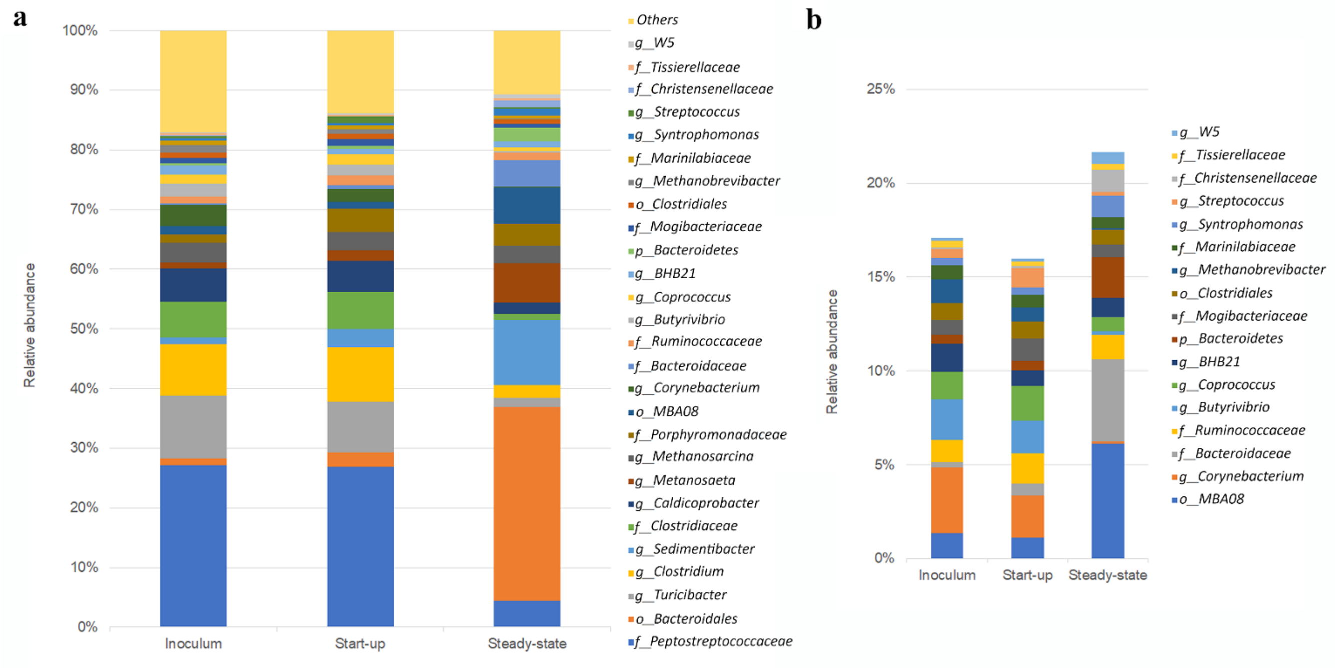

Supplement: Supplementary file 1 — Additional file 1: Table S1. Characteristics of the substrates used as feedstock in the anaerobic digestion tests. Fig. S1 Taxonomic assignments at the genus level. a Bar-plot of the OTU relative abundances in the inoculum and reactor digestate at the start-up and steady state conditions. b Widening of the minor genera. [file 13568_2020_1040_MOESM1_ESM.pdf]
